# Time to Stop Focusing on Catch-Up Growth

**DOI:** 10.1016/j.advnut.2026.100695

**Published:** 2026-07-02

**Authors:** Jef L Leroy, Edward A Frongillo

**Affiliations:** 1 Nutrition, Diets, and Health Unit, International Food Policy Research Institute, Washington, DC, United States; 2 Arnold School of Public Health, University of South Carolina, Columbia, SC, United States

In the late 19th century, Francis Galton observed that very tall parents tended to have tall children but that the children were less tall than the parents themselves. Likewise, very short parents typically had short children, but they were not as short as their parents. He concluded that the height of children selected on the (extreme) height of their parents had moved closer to the population mean [1]. Galton’s “regression toward mediocrity” is now referred to as regression to the mean (RTM).

RTM is deceptively simple. An example explains it in its easiest form. Imagine a group of 200 healthy children of the same age and sex whose height is assessed and recorded independently and within minutes by 2 experienced enumerators. The observed heights for each individual child will slightly differ because of measurement error, but the overall distribution—including the mean and SD—will be the same for each enumerator. If we take the 20 shortest children based on the measurements of the first enumerator, compute their average height, and then recalculate that average using the second enumerator’s measurements, the mean will rise—moving closer to the population mean. The same is observed when starting with the data collected by the second enumerator or when selecting the tallest children. The underlying mechanism is straightforward: extreme values are partly extreme because of random (measurement) error. As neither the direction nor the magnitude of this error is repeated, the next measurement for these children with extreme values tends to be less extreme. RTM occurs only when an individual or sample has been selected based on extreme measurement [2]. RTM does not depend on time: the extremely tall (short) children in Galton’s work will have parents who are not as tall (not as short).

A common practice in nutrition research in low- and middle-income settings is to take repeated height measurements from a group of children to monitor changes over time or to evaluate the impact of an intervention in the absence of a control arm not receiving the intervention. When analyses are restricted to the lower tail of the height distribution, RTM can easily lead to the erroneous conclusion that the status of these children improved or that an intervention had a positive effect when it did not.

The article by Roth et al. [3] in this issue of *Advances in Nutrition* focuses on how RTM complicates the assessment of catch-up growth when defined as stunting reversal. The authors show that RTM is commonly ignored in studies, which may have led to the incorrect conclusion that catch-up growth was present when instead, the apparent improvements in linear growth were (partially or wholly) due to RTM. In addition to the problem of RTM, Roth et al. [3] provide other arguments against the use of stunting reversal or recovery to define catch-up growth. Poor diets and health affect the growth of most children in many low- and middle-income settings. The prevalence of stunting is a way to characterize these population effects but provides no meaningful information about individual children. In addition, children elsewhere in the height-for-age *z*-score (HAZ) distribution may experience improvements in height that remain unnoticed because they do not cross the arbitrary –2 SD cut-off.

There are 3 other reasons to avoid the use of stunting reversal. First, HAZ are constructed using cross-sectional SDs and are therefore unsuitable to evaluate changes in height with age [4,5]. Second, the cross-sectional SDs used in the HAZ denominator increase with age. When the accumulation of linear height deficit with age is smaller than the corresponding increase in SD, centile crossing will occur, suggesting catch-up growth although the child’s status in absolute terms has got worsened. Third, a claim of catch-up growth requires, by definition, a demonstration that an insult occurred that knocked children off their growth trajectory, followed by an event that allowed children to return to that trajectory by having higher than normal growth velocity [6]. In sum, even in the absence of RTM, reversal of stunting is not a meaningful outcome to be studied.

In the second part of their article, Roth et al. [3] claim that RTM could also affect the validity of population-level changes over time, such as changes in the estimated prevalence of stunting. The rationale they present is that study populations, such as those included in Demographic and Health Surveys, are drawn from an underlying distribution of populations with a grand mean. The claimed existence of population-level RTM implies that the mean prevalence of stunting across a group of surveys that are selected based on a high stunting prevalence would be lower when the surveys were reconducted shortly after. Roth et al. [3] neither provide empirical evidence nor any insights as to the magnitude of the purported problem, that is, to what extent it may invalidate global monitoring of malnutrition rates. Furthermore, the authors provide no explanation of the possible mechanisms that could cause population-level RTM.

The decades-old focus in the literature on catch-up growth is implicitly motivated by the belief that improvements in linear growth either are inherently beneficial to children or will automatically lead to improvements in other domains such as child development and long-term health. On the basis of current understanding of mechanisms, neither motivation is a realistic expectation [7]. Growth faltering is a marker of poor conditions in which children are living. Delayed child development and long-term health problems result from these poor conditions. Meaningful improvements in domains such as development and health require interventions that address the underlying drivers of these domains. Rather than investing in demonstrating stunting reversal or catch-up growth in a few children, the nutrition and development communities should focus on how to best improve the conditions in which children grow and develop because poor conditions affect all children. Careful testing of the effectiveness of innovative interventions remains critically important. Evaluations need to assess the intended outcomes and immediate and underlying determinants that adequately capture children’s social and physical environment in which children live [8]. These studies require the use of rigorous methods, including those that avoid spurious findings due to RTM.

## Declaration of Generative AI and AI-assisted technologies in the writing process

The authors declare that no generative AI or AI-assisted technologies were used in the writing of this manuscript.

## Funding

The authors reported no funding received for this study.

## Conflict of interest

The authors report no conflicts of interest.
